# Acurácia da Redução do Segmento-ST Pós-Trombólise como Preditor de Reperfusão Adequada em Estratégia Fármaco-Invasiva

**DOI:** 10.36660/abc.20200241

**Published:** 2021-07-15

**Authors:** Henrique Tria Bianco, Rui Povoa, Maria Cristina Izar, Braulio Luna, Flavio Tocci Moreira, Edson Stefanini, Henrique Andrade Fonseca, Adriano Henrique Pereira Barbosa, Claudia Maria Rodrigues Alves, Adriano Mendes Caixeta, Iran Gonçalves, Pedro Ivo de Marqui Moraes, Renato Delascio Lopes, Angelo Amato Vincenzo de Paola, Dirceu Almeida, Valdir Ambrosio Moises, Francisco A. H. Fonseca

**Affiliations:** 1 Escola Paulista de MedicinaUniversidade Federal de São PauloSão PauloSPBrasilEscola Paulista de Medicina da Universidade Federal de São Paulo, São Paulo, SP - Brasil; 2 Universidade Federal de Sao PauloSão PauloSPBrasilUniversidade Federal de Sao Paulo, São Paulo, SP – Brasil; 3 Hospital Israelita Albert EinsteinSão PauloSPBrasilHospital Israelita Albert Einstein, São Paulo, SP - Brasil; 4 Duke University HospitalDurhamNorth CarolinaEUADuke University Hospital, Durham, North Carolina – EUA

**Keywords:** Intervenção Coronária Percutânea/métodos, Infarto do Miocárdio, Angiografia Coronária, Terapia Trombolítica, Eletrocardiografia/métodos, Dor no Peito, Reperfusão Miocárdica

## Abstract

**Fundamento:**

A intervenção coronária percutânea primária é considerada o “padrão-ouro” para reperfusão coronária. Entretanto, quando não disponível, a estratégia fármaco-invasiva é método alternativo, e o eletrocardiograma (ECG) tem sido utilizado para identificar sucesso na reperfusão.

**Objetivos:**

Nosso estudo teve como objetivo examinar alterações no segmento-ST pós-lise e seu poder de prever a recanalização, usando os escores angiográficos TIMI e *blush* miocárdio (MBG) como critério de reperfusão ideal.

**Métodos:**

Foram estudados 2.215 pacientes com infarto agudo do miocárdio com supra-ST submetidos à fibrinólise [(Tenecteplase)-TNK] e encaminhados para angiografia coronária em até 24 h pós-fibrinólise ou imediatamente encaminhados à terapia de resgate. O ECG foi realizado pré-TNK e 60 min-pós. Os pacientes foram categorizados em dois grupos: aqueles com reperfusão ideal (TIMI-3 e MBG-3) e aqueles com reperfusão inadequada (fluxo TIMI <3). Foi definido o critério de reperfusão do ECG pela redução do segmento ST >50%. Consideramos p-valor <0,05 para as análises, com testes bicaudais.

**Resultados:**

O critério de reperfusão pelo ECG apresentou valor preditivo positivo de 56%; valor preditivo negativo de 66%; sensibilidade de 79%; e especificidade de 40%. Houve fraca correlação positiva entre a redução do segmento-ST e os dados angiográficos de reperfusão ideal (r = 0,21; p <0,001) e baixa precisão diagnóstica, com AUC de 0,60 (IC-95%; 0,57-0,62).

**Conclusão:**

Em nossos resultados, a redução do segmento-ST não conseguiu identificar com precisão os pacientes com reperfusão angiográfica apropriada. Portanto, mesmo pacientes com reperfusão aparentemente bem-sucedida devem ser encaminhados à angiografia brevemente, a fim de garantir fluxo coronário macro e microvascular adequados.

## Introdução

Embora a intervenção coronária percutânea primária (ICP) seja considerada tratamento “padrão-ouro” para o infarto agudo do miocárdio com elevação do segmento-ST (IAMCSST), ela não está suficientemente disponível, principalmente nos países em desenvolvimento.^1^ Considerando esse cenário, a estratégia fármaco-invasiva, com fibrinólise e encaminhamento para angiografia coronária, provou ser opção viável, como orientado por diretrizes e observado em vários estudos, dentre eles o STREAM, com inquestionável benefício quando aplicada nas primeiras horas do evento.^2-5^ Desta forma, em projeto iniciado em 2010, a Secretaria Municipal de Saúde, a Universidade Federal de São Paulo e o Serviço Móvel de Assistência Médica organizaram um sistema planejado de trombólise em núcleos periféricos de saúde, com transferência a centro universitário para a realização de angiografia e tratamento da artéria culpada. Dentro deste contexto, o uso de biomarcadores à beira-leito é essencial para a discriminação de pacientes que obtiveram resultados adequados e aqueles que devem ser encaminhados para a angioplastia de resgate.

O eletrocardiograma (ECG) é método acessível na avaliação dos pacientes com dor torácica, não apenas para fins diagnósticos, mas também na avaliação estratificadora. Desta forma, tem sido proposta a observação do comportamento do segmento-ST como preditor de sucesso terapêutico pós-trombólise na aferição da reperfusão.^6^Portanto, nosso estudo teve como objetivo examinar as modificações do segmento ST pós-lise e seu poder preditivo, tendo como variável de desfecho os escores angiográficos *Thrombolyis in Myocardial Infarction* (TIMI-*flow)* e o *Myocardial Blush Grade* (MBG) como critérios de reperfusão macro e microvascular adequados.

## Métodos

**Figura 1:**
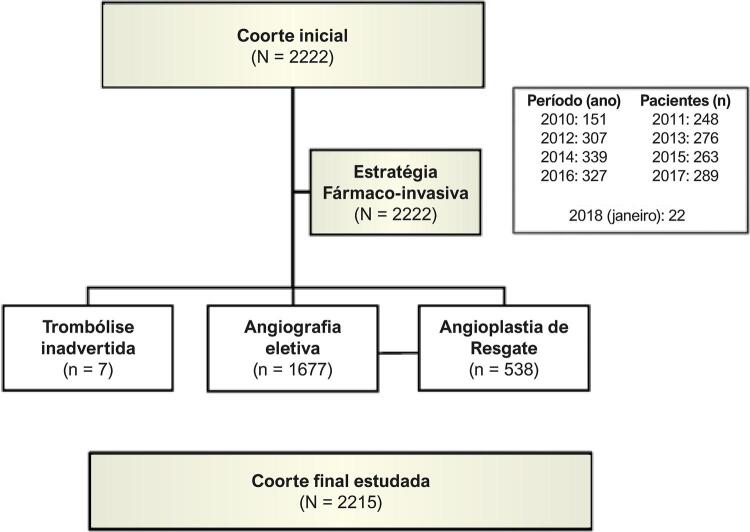
– Fluxograma da coorte estudada.

Estudo transversal, com análise retrospectiva das variáveis de interesse. No período (março de 2010 a janeiro de 2018), 2.215 pacientes foram incluídos consecutivamente e submetidos à terapia trombolítica com tenecteplase (TNK) nos centros primários de saúde, com confirmação eletrocardiográfica do IAMCSST e com encaminhamento para angiografia em até 24 h pós-fibrinólise, ou imediatamente se a terapia de resgate fosse necessária. Dispomos de banco de dados centralizado utilizado para o presente estudo, contendo informações demográficas, clínicas, ECG, tratamentos, intervalos de tempo e eventos hospitalares. O estudo está em conformidade com a Declaração de Helsinque, e o comitê de ética nomeado localmente aprovou o protocolo de pesquisa, sendo o consentimento informado obtido dos pacientes ou de seus representantes legais. O estudo está inscrito e registrado no *ClinicalTrials.gov*, sob número (NCT02090712). O fluxograma da coorte estudada é mostrado na Figura 1.

### Estratégia fármaco-invasiva

A estratégia fármaco-invasiva foi definida como o uso de trombólise com TNK, em dose ajustada pelo peso, seguida de cateterismo cardíaco em até 24 h.^7^Após os resultados do estudo STREAM,^3^em 2013, pacientes >75 anos de idade receberam meia dose (1/2-TNK). Apenas pacientes com contraindicação absoluta à fibrinólise foram excluídos dessa análise. Os pacientes foram pré-medicados com dose de ataque de ácido acetilsalicílico e de clopidogrel. A angiografia de resgate foi indicada por julgamento da equipe médica local, devido à suspeita de trombólise ineficaz para tratamento da artéria relacionada ao infarto (ARI).

### Variável eletrocardiográfica estudada

O critério eletrocardiográfico para reperfusão bem-sucedida foi definido pela redução >50% do supradesnivelamento do segmento ST na derivação com a maior elevação. Os ECG foram realizados pré e 60 min pós-lise e obtidos em 12 derivações (velocidade 25 mm/s; 10 mm/mV). A elevação do ST foi definida como a elevação do ponto J em 2 ou mais derivações contíguas, com limite de ≥0,2 mV nas derivações V1-V3, e ≥0,1mV nas demais derivações. A análise dos segmentos ST foi realizada retrospectivamente e por observadores independentes que desconheciam as características clínicas e angiográficas dos pacientes.

### Variáveis angiográficas estudadas

Cardiologistas intervencionistas experientes realizaram as análises angiográficas de acordo com os escores TIMI-fluxo e MBG descritos a seguir:

- ***Thrombolysis in Myocardial Infarction*** (TIMI-fluxo): **grau 0:** obstrução completa da ARI; **grau 1**: o contraste penetra além do ponto de obstrução, não opacificando completamente o vaso; **grau 2**: opacificação em todo o vaso, porém com fluxo retardado; **grau 3**: há perfusão plena na ARI, com fluxo normal.
- **Myocardial Blush Grade **(MBG): **grau 0**: opacificação do miocárdio na região irrigada pela ARI; **grau 1**: o contraste penetra lentamente na microcirculação e permanece até a injeção de contraste subsequente; **grau 2**: opacificação do miocárdio e saída lenta do contraste, persistindo até o final da fase de *washout*, mas não até a próxima injeção; **grau 3**: fluxo normal na microcirculação, com presença de mínima quantidade ou ausência de contraste na fase de *washout*.

Os pacientes foram categorizados de acordo com o TIMI-fluxo e MBG em dois grupos: aqueles com reperfusão adequada (fluxo TIMI-3 e MBG-3) e aqueles sem reperfusão ótima (fluxo TIMI <3). Aspectos intrínsecos ao procedimento – tais como via de acesso, administração de inibidores de glicoproteína IIb/IIIa e tromboaspiração – ficaram a critério dos operadores médicos. As análises das angiografias foram realizadas retrospectivamente por investigadores independentes, e que desconheciam as características clínicas e epidemiológicas dos pacientes incluídos no estudo.

### Análise estatística

Variáveis contínuas foram descritas como média ± desvio padrão (m ± dp) ou mediana e intervalo interquartil, md (IIQ), conforme normalidade dos dados. Para a avaliação dos pressupostos de normalidade, utilizamos o teste de Kolmogorov-Smirnov e a inspeção dos coeficientes de curtose e assimetria. As variáveis categóricas foram descritas por distribuições de frequência absoluta e relativa. Para as comparações entre os grupos, utilizamos Teste-T de Student não pareado ou Teste-U não paramétrico de Mann-Whitney para variáveis contínuas e teste qui-quadrado (χ2) de Pearson para variáveis categóricas, com correção de Yates. O coeficiente de Kendall-tau, teste de hipótese não paramétrico, foi utilizado para verificar a correlação entre a redução do segmento ST e os padrões angiográficos em variáveis categóricas dicotômicas. Curvas ROC (*receiver operating characteristic curve) *foram construídas para a inspeção de sensibilidade/especificidade da regressão do segmento ST com base nos escore TIMI-fluxo e MBG. A razão de verossimilhança positiva [(*positive likelihood-ratio*), RV (+)] foi calculada: RV (+) = sensibilidade / (1- especificidade). Bons testes de diagnóstico têm RV (+) >10, e seu resultado positivo tem contribuição significativa. A razão de verossimilhança para resultados negativos [(*negative likelihood-ratio*), RV (-)] é um bom indicador para descartar o diagnóstico. Bons testes diagnósticos têm RV (-) <0,1; calculada: RV (-) = (1- sensibilidade) / especificidade. A razão de chance diagnóstica, [(*diagnostic odds ratio*), RCD] também é uma medida de precisão, usada para estimar o poder discriminativo e a comparação de precisões entre os testes. A RCD foi a razão das chances de positividade no grupo com redução do segmento ST >50% com as chances de positividade no grupo sem redução ST. Consideramos p-valor <0,05 estatisticamente significante *a priori* para todas as análises, com testes bicaudais. As análises foram realizadas no SPSS-versão-20 (*IBM-SPSS Statistics, New York, EUA*)^®^.

## Resultados

Foram incluídos pacientes de ambos os sexos, sendo 70,2% do sexo masculino; a mediana da idade foi de 58 anos, IIQ (50-66). O tempo desde o início dos sintomas até o centro de atenção primária foi de 220 min, IIQ (140-345). A prevalência de fatores de risco e as características dos pacientes no período basal são relatadas na Tabela 1. Em resumo, 60,7% eram hipertensos; 29,8% tinham diabetes; 63,1% eram fumantes. Além disso, 11% apresentavam infarto prévio do miocárdio, e 4,3%, acidente vascular encefálico prévio. Os preditores de risco utilizados foram registrados no período basal. Observamos que a maioria dos pacientes apresentava-se em classe funcional baixa pela classificação Killip-Kimball:^8^**I** (73%), **II** (16,3%), **III** (2,2%), **IV** (8,6%); e com baixo perfil de risco pelos escores de predição, TIMI-*Risk*:^9^ 3, IIQ (2-5); GRACE^10^ 136, IIQ (117-161), ou seja, perfil de baixo risco.

**Tabela 1:** – Características da coorte estudada no período basal

Variáveis	Todos os pacientes (N = 2215)	Pacientes Com redução ST (n = 1511)	Pacientes sem redução ST (n = 704)	Valor de p
Demográficas				
Idade, anos	58 (50-66)	58 (50-66)	58 (49-66)	0,62
Sexo masculino	70,2%	70,5%	69,6%	0,41
Índice de massa corporal, kg/m^2^	26 (24-29)	26 (24-29)	26 (24-30)	0,004
**Características hemodinâmicas**				
Pressão sistólica, mmHg, MD (IIQ)	130 (115-150)	130 (116-150)	130 (110-150)	0,098
Pressão diastólica, mmHg, MD (IIQ)	80 (70-93)	80 (70-94)	80 (70-93)	0,23
Frequência cardíaca, bpm, MD (IIQ)	76 (66-90)	75 (66-88)	80 (68-95)	<0,001
Fatores de risco				
Hipertensão, (%)	60,7%	59,2%	64,5%	0,01
Diabetes melito, (%)	29,8%	28,1%	33,5%	0,01
Tabagistas atuais, (%)	63,1%	66,1%	57,5%	<0,001
Infarto prévio do miocárdio, (%)	11%	9,9%	4,9%	0,02
AVE* prévio, (%)	4,3%	4,2%	4,1%	0,98
eGFR^**†**^, mL/min/1,73 m^2^, MD (IIQ)	86 (67-108)	88 (70-110)	84 (63-106)	0,015
**Escores de risco**				
TIMI-*risk *escore, MD (IIQ)	3 (2-5)	3 (2-5)	4 (2-6)	<0,001
GRACE-*risk* escore, MD (IIQ)	136 (117-161)	134 (118-157)	140 (115-171)	0,004
**Tempos pivotais**				
Tempo dor-agulha, MD (IIQ), min.	220 (140-345)	220 (145-345)	220 (140-356)	0,34
Tempo porta-agulha, MD (IIQ), min.	75 (45-135)	74 (45-130)	75 (44-150)	0,057
Tempo TNK^**‡**^-cate, MD (IIQ), min.	740 (335-1380)	960 (405-1440)	410 (270-841)	<0,001

Os dados sobre histórico médico, comorbidades e tempo para a apresentação foram amplamente derivados de entrevista médica. As informações demográficas e fatores de risco foram reportadas pelos pacientes e equipe treinada reviu os dados durante a admissão hospitalar. Para as comparações entre grupos, utilizamos o teste não paramétrico de Mann-Whitney para variáveis contínuas e testes de χ2 para variáveis discretas. Os dados são expressos como mediana (MD) e intervalo interquartil (IIQ), ou número e porcentagem. AVE*: acidente vascular encefálico; eGFR^†^: taxa de filtração glomerular estimada por MDRD (Modification of Diet in Renal Disease); TNK^‡^: tenecteplase; cate: laboratório de cateterismo.

### Distribuição do fluxo coronário de acordo com o padrão TIMI/MBG

Na avaliação subjetiva do fluxo TIMI e MBG, a concordância interobservadores foi de 94%. Distribuição da ARI de acordo com o escore TIMI-fluxo (0-3): 21%; 3,6%; 14,4%; 61%, respectivamente. Avaliação do MBG (0-3): 42%; 3,5%; 2,5%; 52%, respectivamente, avaliadas apenas em pacientes com fluxo TIMI-3.

### Distribuição das artérias relacionadas ao infarto

Observamos a seguinte distribuição das ARI: artéria descendente anterior esquerda (40,3%); coronária direita (35,3%); coronária circunflexa esquerda (6,8%); e ramos das principais artérias (17,6%). A descrição das ARI e a análise das regiões envolvidas no ECG e sua distribuição, de acordo com o sexo, podem ser vistas na Tabela 2.

**Tabela 2:** – Descrição da artéria relacionada ao infarto e análise das regiões eletrocardiográficas com elevação do segmento-ST e sua distribuição de acordo com o sexo

**Artéria relacionada ao infarto**	**Todos os pacientes, N (%)**	**Homens (%)**	**Mulheres (%)**
Artéria descendente anterior	893 (40,3)	41,9	36,4
Artéria coronária direita	782 (35,3)	33,5	39,6
Artéria coronária circunflexa	151 (6,8)	6,5	7,6
Artéria coronária esquerda (tronco)	11 (0,5)	0,6	0,3
Artéria coronária descendente posterior	8 (0,36)	0,3	0,5
Arterária coronária ventricular posterior	14 (0,63)	0,6	0,6
Artéria coronária marginal esquerda	27 (1,21)	1,2	1,4
Artéria ramo diagonal	15 (0,67)	0,6	0,9
Artéria não identificada ou outras	335 (15,2)	15,4	13,1
**Elevação do segmento-ST (ECG)**	**Todos os pacientes N (%)**	**Homens (%)**	**Mulheres (%)**
Parede anterosseptal	892 (40,3)	41,9	36,4
Parede anterior	782 (35,3)	33,5	39,6
Parede anterior extensa	151 (6,8)	6,5	7,6
Parede inferior	12 (0,5)	0,6	0,3
Parede inferolateral	49 (2,2)	2,1	2,4
Parede lateral	15 (0,7)	0,6	0,9
Não identificada ou outras	314 (14,2)	14,8	4,7

*Os dados são expressos em frequência e porcentagem (%). Uma equipe de especialistas independentes revisou os achados eletrocardiográficos. A concordância entre pares de observação e o índice Kappa foi utilizada como medida de variabilidade. Os revisores de ECG desconheciam as características basais dos pacientes. A elevação do segmento ST foi medida 20 ms após o ponto J. O infarto foi considerado: anterosseptal (V1-V4); anterior: (V1-V6); anterior extenso: (V1-V6 + DI, aVL); inferior: (DII, DIII, aVF); inferolateral: (DII, DIII, aVF + V5-V6); lateral: (V5-V6). A artéria relacionada ao infarto foi identificada de acordo com a presença de trombo, oclusão total ou retardo do fluxo anterógrado.*

### Distribuição das medidas pelo critério ECG de acordo com o fluxo TIMI/MBG

**Figura 2:**
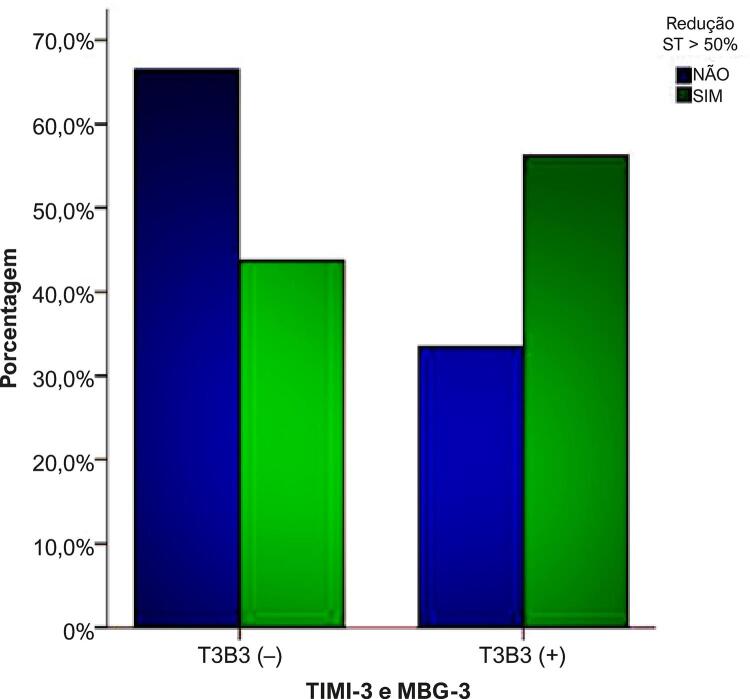
– Correlação entre os padrões angiográficos (TIMI-3 e MBG-3) e o critério eletrocardiográfico de reperfusão.

**Figura 3:**
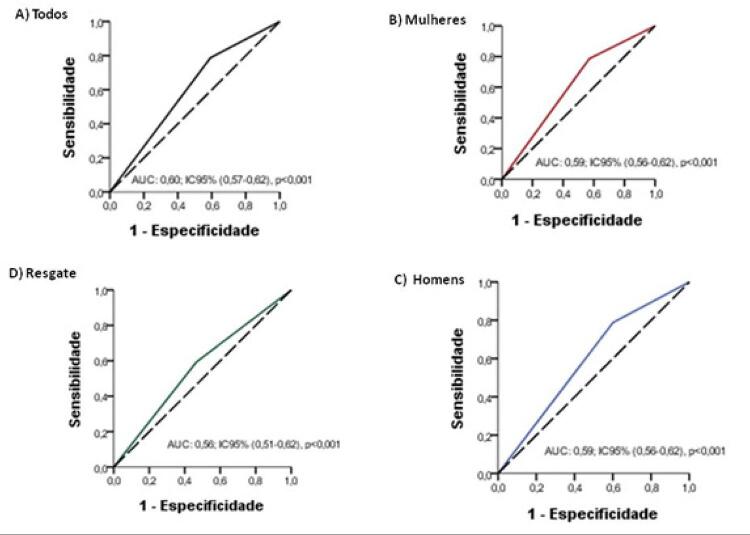
– Área sob a curva em pacientes que apresentaram TIMI-fluxo -3/MBG- 3 e redução do segmento ST pós-trombólise. Todos os pacientes, grupo resgate e estratificado pelo sexo.

A predição de reperfusão coronária adequada utilizando o critério ECG (redução do segmento ST >50%) mostrou valor preditivo positivo de 56% [IC 95% (0,54-0,59)]; valor preditivo negativo de 66% [IC 95% (0,62-0,70)]; sensibilidade de 79% [IC 95% (0,76-0,81)]; e especificidade de 40% [IC 95% (0,38-0,44)]. Razão de verossimilhança positiva (RV +) de 1,32; razão de verossimilhança negativa (RV –) de 0,52. A razão de chance diagnóstica [RCD (*diagnostic odds ratio*)] foi de 2,55 (razão entre chances de positividade no grupo com redução do segmento ST em relação às chances de positividade no grupo sem redução ST). Assim, observamos correlação positiva fraca entre a redução do segmento ST e o padrão angiográfico de reperfusão, considerando TIMI-3 e MBG-3, (r = 0,21; p <0,001) como observado na Figura 2. Destacamos, ainda, o comportamento do critério ECG para recanalização e sua correspondência com os escores angiográficos, estratificados por sexo, que não foram significativamente diferentes (Tabela 3). A Figura 3 mostra a área sob a curva (AUC) de 0,60 [IC-95% (0,57-0,62)] naqueles com redução do segmento ST e o padrão angiográfico (TIMI-3/MBG-3): [(**A**-geral; **B**-feminino; **C**-masculino)].

**Tabela 3:** – Correlação entre redução do segmento ST e os escores TIMI- 3/Blush- 3, estratificados por sexo

Coeficiente de correlação (índice de Kendall)					
Sexo				Redução do segmento ST	TIMI-3/Blush-3
**Homens**	Coeficiente de Kendall	Redução-ST	Coeficiente de correlação	1,0	0,203^*^
			N	1537	1506
		TIMI- 3/Blush-3	Coeficiente de correlação	0,203^*^	1,0
			N	1506	1523
**Mulheres**	Coeficiente de Kendall	Redução-ST	Coeficiente de correlação	1,0	0,231^*^
			N	652	637
		TIMI- 3/Blush-3	Coeficiente de correlação	0,231^*^	1,0
			N	637	643

* A correlação foi significativa no nível 0,05 (teste bicaudal).

### Características clínicas dos pacientes encaminhados para terapia de resgate

Os pacientes foram encaminhados para a terapia de resgate pelo julgamento da equipe médica local, com taxa de 24,28%. As características clínico-epidemiológicas entre o grupo resgate e o grupo angiografia eletiva podem ser observadas na Tabela 4. No grupo resgate, encontramos sensibilidade e especificidade de 59% e 53%, respectivamente, para a redução do ST na predição de (TIMI-3/MBG-3). A AUC foi de 0,56 [IC-95% (0,51-0,62)] conforme mostrado na Figura 3: [(grupo **D**-resgate)].

**Tabela 4:** – Características clínico-epidemiológicas dos pacientes do grupo angiografia eletiva e dos encaminhados para angioplastia de resgate

Variáveis	Angiografia eletiva (n = 1677)	Angioplastia de resgate (n = 538)	Valor de p
**Demográficas**			
Idade, (anos), m ± dp	58,26 ± 11,61	58,67 ± 11,53	0,47
Sexo masculino, n (%)	1178 (71, 7%)	358 (66,5%)	0,03
**Fatores de risco**			
Infarto prévio do miocárdio, n (%)	218 (11,2%)	57 (10,6%)	0,75
AVE* prévio, n (%)	108 (0,5%)	14 (2,6%)	0,05
Tabagistas atuais, n (%)	589 (35, 9%)	211 (39, 2%)	0,18
Hipertensão arterial, n (%)	678 (41, 3%)	186 (34, 7%)	0,01
Diabetes melito, n (%)	490 (27, 8%)	186 (34, 5%)	0,006
**Variáveis hemodinâmicas**			
PA^**†**^ sistólica, (mmHg), m ± dp	134,3 ± 27,56	130,91 ± 29,65	0,06
PA^**†**^ diastólica, (mmHg), m ± dp	82,78 ± 18,36	81,16 ± 19,50	0,11
Frequência cardíaca, (bpm), m ± dp	78 ± 17,55	81 ± 19,70	0,002

Os dados são expressos como média (m) ± desvio padrão (dp) ou porcentagem (%). Informações clínicas, demográficas e fatores de risco foram revisadas durante a admissão hospitalar. AVE*: acidente vascular encefálico; PA^**†**^: pressão arterial.

## Discussão

Um aspecto crítico da redução dos danos associados ao infarto do miocárdio é a garantia de acesso à emergência, na qual a identificação dos sintomas e a obtenção de ECG inicial são fundamentais. Com ICP indisponível em unidades básicas de saúde e em muitos hospitais, a estratégia fármaco-invasiva é alternativa recomendada no IAMCSST.^2^ O melhor método para obtenção da reperfusão tem sido tema de debate, essencialmente enviesado pela percepção competitiva entre as possibilidades de revascularização. Encontra-se bem fundamentada que a melhor estratégia é aquela que está disponível dentro de prazos bem estabelecidos, sendo indiferentes nas primeiras horas de início dos sintomas isquêmicos. O grupo dos trialistas demonstrou que, entre os pacientes com dor dentro de 6 horas, 30 mortes foram prevenidas/1.000; entre 7 a 12 horas, seriam evitadas aproximadamente 20 mortes/1.000.^11^ Destacam-se, portanto, a importância do rápido reconhecimento dos sintomas isquêmicos e a prontidão no tratamento. Desta forma, tanto a ICP primária quanto a fibrinólise são tratamentos bem estabelecidos, e os benefícios são maximizados quando o tratamento ocorre precocemente.^12^Metanálise relatou risco aumentado de insuficiência cardíaca de início recente, com aumento relativo na incidência por hora de atraso nas estratégias de reperfusão.^13^

Nosso estudo foi uma tentativa de fornecer, por meio da interpretação das alterações no ECG pós-fibrinólise à beira-leito, uma estratificação de risco, determinando quais pacientes deveriam ser encaminhados para estudo angiográfico urgenciado e quais poderiam ser encaminhados de maneira eletiva. De fato, o ECG tem papel fundamental na precisão diagnóstica, devendo ser obtido o mais precocemente possível. No cenário das síndromes agudas, biomarcadores de reperfusão, obtidos à beira-leito, são cruciais na estratificação. Assim, a utilidade do critério de redução do segmento ST em prever a reperfusão tem sido empregada como desfecho substituto em alguns ensaios clínicos. Em análise de subgrupo proveniente da coorte DANAMI-2 (*DANish trial in Acute Myocardial Infarction-2*), a resolução do ST após 4 h associou-se às maiores taxas de reinfarto entre os pacientes que receberam fibrinolítico, enquanto não foi observada nos pacientes que receberam tratamento percutâneo.^14^O tempo para obter ECG pós-fibrinólise é variável. No estudo REACT, foi definida a redução de 50% do ST aos 90 min pós-fibrinólise, já o estudo MERLIN definiu reperfusão coronária usando o mesmo critério aos 60 min. O tempo médio desde o início dos sintomas até o resgate para a ICP foi de 414 min no estudo MERLIN e 327 min no REACT.^15,16^ A reperfusão bem-sucedida foi considerada quando o fluxo distal esteve restaurado na artéria culpada utilizando-se do escore angiográfico TIMI-fluxo, descrito previamente.

Dentro deste contexo, postulou-se a teoria da “artéria aberta”, indicando impacto prognóstico se a artéria fosse recanalizada.^17^ Nesse amplo espectro, Gibson et al.18 introduziram um refinado método na avaliação da perfusão miocárdica, o TIMI-fluxo, onde a reperfusão era considerada angiograficamente bem-sucedida quando o TIMI-3 fosse alcançado.^19,20^ No entanto, mesmo quando alcançado esse escore, alguns pacientes apresentam perfusão tissular inadequada e vários mecanismos têm sido sugeridos – dentre outros, a embolização distal e o fenômeno *no-reflow*.^21-23^Posteriormente, Hoffmann et al.^24^divulgaram o conceito de rubor miocárdio (MBG) como índice angiográfico do fluxo microvascular, posto que pacientes com fluxo TIMI-3 e MBG normal apresentavam menores taxas de mortalidade. A restauração da perviedade vascular coronária não é garantia de perfusão tecidual, portanto, entendemos que a presença de MBG adequado é uma característica prognóstica relevante e deveria ser adicionada à classificação fluxo-TIMI, comumente usada para definir reperfusão angiográfica bem-sucedida.^25-27^Anormalidades avaliadas pelo MBG correlacionam-se com remodelamento ventricular desfavorável, mesmo após ajustes para a presença de fluxo TIMI-3.^28-31^

Utilizada como critério de reperfusão, identificamos ensaios associando a persistência da elevação do segmento ST com prognósticos. Em interessante estudo, foi observado que, em pacientes submetidos a ICP-primária, a redução do ST não previu mortalidade a longo prazo.^32^ Outro estudo não encontrou alteração no valor prognóstico da redução de ST pós-ICP em acompanhamento de longo prazo para os principais desfechos.^33^Por sua vez, o comportamento do segmento-ST dentro de 60 minutos após ICP-primária bem-sucedida também foi avaliado por sua associação com menores taxas de mortalidade em curto e longo prazo, com gradação da redução do ST (>70%; 30-70%; <30%). Neste estudo, na ausência de redução do segmento ST, foram identificados pacientes com menor probabilidade de benefício da restauração precoce do fluxo na ARI, provavelmente devido a danos microvasculares e subsequente menor preservação miocárdica.^34^Todavia, grande parte destes estudos contemplou pacientes submetidos à ICP-primária, e não pós-trombólise, além de não consideraram o padrão MBG como definidor de melhor padrão angiográfico de reperfusão. A presença de resolução precoce da elevação do segmento ST pós ICP-primária angiograficamente bem-sucedida identifica pacientes com maior probabilidade de se beneficiar da restauração precoce do fluxo na ARI. Destaca-se, ainda, que as medidas TIMI-fluxo superestimam muito o sucesso na ICP-primária. Nos estudos pós-fibrinólise, a definição de sucesso angiográfico era caracterizada pela presença de TIMI 2 e 3. Entretanto, pacientes com TIMI-fluxo <3 apresentam pior evolução clínica, sobretudo a médio e longo prazo, quando comparados ao grupo de pacientes com TIMI-fluxo-3.

A medição exata da elevação do segmento ST é complexa e poderia prejudicar a simplicidade do modelo, sobretudo na sala de emergência. No entanto, na maioria dos casos, mensurações precisas não são necessárias, pois podem ser facilmente obtidas em comparação visual dos ECG.^35^Vários métodos para avaliar a resolução de ST são descritos, a maior parte deles inclui a resolução porcentual; alguns utilizam uma única derivação, enquanto outros mensuram a somatória de desvios em múltiplos canais. Em nossos apontamentos, a detecção da reperfusão usando critérios mais rigorosos, como a resolução completa do segmento ST ou redução >70%, apresentou melhor especificidade, porém com baixa sensibilidade e pouca capacidade preditiva. A acurácia prognóstica de distintos métodos na avaliação do segmento ST pós ICP foi analisada no estudo *Controlled Abciximab and Device Investigation to Lower Late Angioplasty Complications* (CADILLAC), demonstrando que a análise do ST em valores absolutos em derivação única no ECG pós-intervenção foi ao menos equivalente, do ponto de vista prognóstico, a algoritmos mais complexos.^36^ A avaliação da reperfusão angiográfica pode ser prevista por alterações no segmento ST e foi descrita em nossa coorte. Quando aplicamos o índice de correlação entre o critério TIMI-3 e MBG-3 e reperfusão, pelo critério de ECG, obtivemos valor semelhante ao observado quando consideramos apenas a classificação angiográfica fluxo TIMI-3. Mesmo agrupando e comparando pacientes com alterações isquêmicas em parede anterior *versus* infarto “não anterior”, a redução do segmento ST >50% não previu de forma acurada os melhores padrões angiográficos.

Embora existam terapias eficazes, ainda há carência de informações qualitativas disponíveis para a estratificação, notadamente no modelo de estratégia fármaco-invasiva, em que a avaliação preliminar em centros e unidades básicas de saúde pode ser impactante a um melhor prognóstico. A utilização de modelos multivariados na forma de escores representa uma forma interessante na predição, superior à obtida de maneira subjetiva apenas pela impressão clínica. Ferramentas que auxiliem a capacidade médica em avaliar rápida e precisamente o risco são, portanto, de grande interesse. Não obstante a existência de preditores bem caracterizados, a estimativa de risco é desafiadora, em virtude de perfis complexos que demandam integração de variáveis e com calibração para populações locais.^37^

Em nossa coorte, registramos 116 óbitos (5,3%) e de maneira esperada, com maior incidência no grupo resgate (11,5% *vs.* 2,4%). Complicações como choque cardiogênico e instabilidades elétricas graves foram as principais causas de mortalidade, com os pacientes pertencentes à classificação funcional IV (Killip-Kimball) com as maiores taxas de letalidade. No modelo de regresão multivariável, a classe Killip-Kimball foi o preditor mais poderoso, com risco crescente de mortalidade a cada piora de categoria. Outro importante ponto a se destacar é que menores taxas de mortalidade foram observadas no grupo que recebeu a trombólise mais cedo, reforçando o conceito de que reperfusão precoce atenua o risco de complicações, pelo menos em curto prazo.

Idealmente, um indicador de reperfusão deve ser prontamente obtido e aplicável principalmente à beira-leito. Em nossa coorte, o encaminhamento para o estudo angiográfico precoce deveu-se possivelmente frente às modificações no segmento ST, pois pacientes que não apresentaram redução pós-trombólise foram referenciados à terapia de resgate mais rapidamente. No grupo com redução do ST >50%, o tempo mediano entre a trombólise e o laboratório de cateterismo foi de 960 min *versus* 410 min no grupo sem redução do ST. Entretanto, essa pode ter sido uma opção equivocada, pois 24,5% apresentavam TIMI-3/MBG-3 caracterizando diagnóstico de gravidade falso-positivo. Além disso, 38,5% do grupo que apresentou redução >50% apresentava padrão angiográfico TIMI-fluxo <3, subestimando o risco real. Em recente estudo de nosso grupo, com a inclusão de 104 pacientes e análise da mensuração do intervalo QT e sua dispersão nas 12 derivações, e também apenas na região com supradesnivelamento do segmento ST (nominada dispersão regional do intervalo QT) pré e 60 min pós-lise, observamos aumento da dispersão regional do intervalo QT (dQT-Reg) pós-lise em infartos de parede anterior nos casos fluxo TIMI-3/MBG-3, com sensibilidade e especificidade de 93% e 71%, respectivamente. Embora com número pequeno de pacientes avaliados, estes dados sugerem a dQT-Reg um instrumento promissor na identificação não invasiva de reperfusão.^38^

É clinicamente reconhecida a capacidade em predição do alívio dos sintomas isquêmicos durante a reperfusão.^39^ No entanto, este relato fica sujeito às influências idiossincráticas, assim como possível mascaramento dos sintomas pela utilização de fármacos (nitratos, analgesia, sedação). Um grave problema identificado em alguns centros é a fibrinólise inadvertida pela leitura incorreta dos ECG, com reportagem de que 5,7% a 11,0% dos pacientes tratados como IAMCSST não apresentavam infarto do miocárdio.^40^ Em nossa coorte, identificamos sete casos que, inadvertidamente, receberam trombolíticos pelas seguintes causas: pericardite aguda, anormalidades não isquêmicas da repolarização ventricular e dissecção da aorta.

A instituição da terapia trombolítica precoce continua sendo pedra angular para melhorar a sobrevida pós-IAMCSST. No entanto, como evidenciado em nossas anotações, o atraso, entre o início dos sintomas até a admissão nas unidades de saúde foi prolongado, não havendo, entretanto, diferenças quanto ao comportamento do segmento ST pós-trombólise. No modelo de regressão multinomial, nossos dados sinalizam para os melhores resultados angiográficos aos pacientes encaminhados precocemente ao estudo hemodinâmico, de forma independente do comportamento do segmento ST. Portanto, acreditamos que a melhor estratégia pós-trombólise é a rápida condução ao centro de angiografia referenciado.^41^ No entanto e desafortunadamente, o tempo disponível para esses pacientes permanece inadequado, possivelmente devido aos sérios problemas logísticos enfrentados nas grandes cidades. Por esse motivo, reduzir esses tempos continua sendo um desafio em projetos de saúde pública.^42^

### Limitações do estudo

Limitações do presente estudo devem ser declaradas. Esta foi uma análise exploratória e de centro único. Além disso, os ECG e as angiografias foram analisadas de forma retrospectiva. Nas redes clínicas, a estratificação de risco é apenas um dos parâmetros que determinam atraso para a angiografia. No estudo STREAM,^3^ citado previamente, o tempo médio para angiografia foi de 2,2 h para pacientes que necessitaram de intervenção urgente, e 17 h para os 64% restantes. No entanto, nossos achados apontam para tempos mais altos, sobretudo para o grupo-resgate, presumivelmente face às dificuldades na transferência para o centro de hemodinâmica (ambulância não disponível imediatamente). Todavia, indicam a expressão pragmática do atendimento público em uma grande cidade. Como último ponto a ser destacado, nosso estudo não testou especificamente o desempenho do ECG para reperfusão, mas analisou o critério de ECG recomendado por diretrizes.

## Conclusão

Nossos resultados sugerem que a redução do segmento ST, analisada conforme recomendado, não conseguiu identificar com precisão os pacientes com reperfusão angiográfica adequada, com base nos escores TIMI-fluxo e MBG. Acreditamos, portanto, que o estudo angiográfico precoce oferece a oportunidade na identificação de falhas na fibrinólise. Desta forma, na ausência de melhores biomarcadores, mesmo pacientes com reperfusão aparentemente bem-sucedida devem ser encaminhados para o laboratório de hemodinâmica o mais breve possível, vislumbrando garantir fluxo coronário adequado, considerando os aspectos macro e microvasculares.
