# Supplementary material for: Acurácia da Redução do Segmento-ST Pós-Trombólise como Preditor de Reperfusão Adequada em Estratégia Fármaco-Invasiva
Source: Arq Bras Cardiol. 2021 Jul 15;117(1):15–25. [Article in Portuguese] doi: 10.36660/abc.20200241 (PMC8294746; doi:10.36660/abc.20200241)
Supplement: Translation

# Translation: Accuracy of Post-thrombolysis ST-segment Reduction as an Adequate Reperfusion Predictor in the Pharmaco-Invasive Approach

**Authors:** Henrique Tria Bianco, Rui Povoa, Maria Cristina Izar, Braulio Luna, Flavio Tocci Moreira, Edson Stefanini, Henrique Andrade Fonseca, Adriano Henrique Pereira Barbosa, Claudia Maria Rodrigues Alves, Adriano Mendes Caixeta, Iran Gonçalves, Pedro Ivo de Marqui Moraes, Renato Delascio Lopes, Angelo Amato Vincenzo de Paola, Dirceu Almeida, Valdir Ambrosio Moises, Francisco A. H. Fonseca

**Affiliations:** 1 BrasilEscola Paulista de Medicina da Universidade Federal de São Paulo, São Paulo, SP - Brasil; 2 BrasilUniversidade Federal de Sao Paulo, São Paulo, SP – Brasil; 3 BrasilHospital Israelita Albert Einstein, São Paulo, SP - Brasil; 4 EUADuke University Hospital, Durham, North Carolina – EUA

**Keywords:** Percutaneous Coronary Intervention, Myocardial Infarction, Coronary Angiography, Thrombolytic Therapy, Electrocardiography/methods, Chest Pain, Myocardial Reperfusion

## Abstract

**Background:**

Primary percutaneous coronary intervention is considered the “gold standard” for coronary reperfusion. However, when not available, the drug-invasive strategy is an alternative method and the electrocardiogram (ECG) has been used to identify reperfusion success.

**Objectives:**

Our study aimed to assess ST-Segment changes in post-thrombolysis and their power to predict recanalization and using the angiographic scores TIMI-flow and Myocardial Blush Grade (MBG) as an ideal reperfusion criterion.

**Methods:**

2,215 patients with ST-Segment Elevation Myocardial Infarction (STEMI) undergoing fibrinolysis [(Tenecteplase)-TNK] and referred to coronary angiography within 24 h post-fibrinolysis or immediately referred to rescue therapy were studied. The ECG was performed pre- and 60 min-post-TNK. The patients were categorized into 2 groups: those with ideal reperfusion (TIMI-3 and MBG-3) and those with inadequate reperfusion (TIMI and MBG <3). The ECG reperfusion criterion was defined by the reduction of the ST-Segment >50%. A p-value <0.05 was considered for the analyses, with bicaudal tests.

**Results:**

The ECG reperfusion criterion showed a positive predictive value of 56%; negative predictive value of 66%; sensitivity of 79%; and specificity of 40%. There was a weak positive correlation between ST-Segment reduction and ideal reperfusion angiographic data (r = 0.21; p <0.001) and low diagnostic accuracy, with an AUC of 0.60 (95%CI: 0.57-0.62).

**Conclusion:**

The ST-Segment reduction was not able to accurately identify patients with adequate angiographic reperfusion. Therefore, even patients with apparently successful reperfusion should be referred to angiography soon, to ensure adequate macro and microvascular coronary flow.

## Introduction

Although primary percutaneous coronary intervention (PCI) is considered the “gold standard” treatment for ST-Segment elevation myocardial infarction (STEMI), it is not always available, especially in developing countries.^1^ Considering this scenario, the drug-invasive strategy, with fibrinolysis and referral for coronary angiography, has proved to be a viable option, according to the guidelines and observed in several studies, including STREAM, with unquestionable benefits when applied within the first hours of the event.^2-5^ Therefore, a project was started in 2010, by the Municipal Health Department, Universidade Federal de Sao Paulo and the Itinerant Health Care Service, which organized a planned system of thrombolysis in suburban health centers, including transfer to the university center for angiography and treatment of the related artery. Hence, the use of bedside biomarkers is essential for the discrimination of patients who have obtained adequate results and those who should be referred for rescue angioplasty.

The electrocardiogram (ECG) is an accessible method to evaluate patients with chest pain, not only for diagnostic purposes, but also in the stratification assessment. Thus, observing the behavior of the ST-Segment as a predictor of post-thrombolysis therapeutic success in reperfusion has been proposed.^6^Therefore, our study aimed to assess the modifications of the post-fibrinolysis ST-Segment and its predictive power, with the angiographic scores “Thrombolysis in Myocardial Infarction” (TIMI-flow) as the outcome variable and the “Myocardial Blush Grade” (MBG) as the appropriate macro and microvascular reperfusion criteria.

## Methods

This was a cross-sectional study, with retrospective analysis of the variables of interest, carried out from March-2010 to January-2018. A total of 2,215 patients were consecutively enrolled and submitted to thrombolytic therapy with tenecteplase (TNK) in primary health centers, with electrocardiographic confirmation of STEMI and referred for angiography within 24 hours post-fibrinolysis, or immediately if rescue therapy was necessary. A centralized database was used for the present study. It contained demographic, and clinical information, ECG results, treatments, time intervals and hospital events. This study is in accordance with the Declaration of Helsinki, was approved by the local Ethics Committee, and the free and informed consent was obtained from patients or their legal representatives. The study is registered in the *ClinicalTrials.gov *database, under number NCT02090712. The study flowchart is shown in Figure 1.

**Figure 1:** – Flowchart of the studied cohort.

### Drug-invasive strategy

The drug-invasive strategy was defined as the use of thrombolysis with TNK, using a weight-adjusted dose , followed by cardiac catheterization within 24 hours.^7^After the STREAM ^3^study results in 2013, patients aged >75 years received half a dose (1/2-TNK). Only patients with an unquestionable contraindication to fibrinolysis were excluded from this analysis. The patients were premedicated with a loading dose of acetylsalicylic acid and clopidogrel. Rescue angiography was indicated by the local medical staff due to the suspected ineffective thrombolysis for the treatment of the infarct-related artery (IRA).

### Assessed electrocardiographic variable

The electrocardiographic criterion for successful reperfusion was defined by the reduction >50% of ST-Segment elevation in the lead with the highest elevation. The ECGs were performed pre-and 60-minutes post-fibrinolysis and obtained in 12 leads (velocity 25 mm/s; 10 mm/mV). The ST-Elevation was defined as the elevation of the J-point in 2 or more contiguous leads, with a limit of ≥0.2 mV in the V1-V3 and ≥0.1mV in the other leads. The analysis of the ST-Segment was performed retrospectively and by independent observers who were unaware of the patients’ clinical and angiographic characteristics.

### Assessed angiographic variables

Experienced interventionist cardiologists performed the angiographic analyses according to the TIMI-flow and MBG scores described below:

- **Thrombolysis in Myocardial Infarction** (TIMI-flow):**grade 0:** complete obstruction of IRA; **grade 1**: contrast penetrates beyond the point of obstruction, not completely opacifying the vessel; **grade 2:** opacification throughout the vessel, but with delayed flow; **grade 3:** full perfusion in the IRA, with normal flow.
- **Myocardial Blush Grade** (MBG): **grade 0:** no myocardial blush or contrast density; **grade 1:** minimal myocardial blush or contrast density; **grade 2**: moderate myocardial blush or contrast density but less than that obtained during angiography of a contralateral or ipsilateral non–infarct-related coronary artery; and **grade 3**: normal myocardial blush or contrast density, comparable with that obtained during the angiography of a contralateral or ipsilateral non–infarct-related coronary artery.

The patients were categorized according to the TIMI-flow and MBG into two groups: those with adequate perfusion (TIMI-3 and MBG-3 flow) and those without optimal reperfusion (TIMI flow <3). Intrinsic aspects to the procedure, such as access route, administration of glycoprotein IIb/IIIa inhibitors and thrombus-aspiration, were a medical team choice. The analyses of the angiography were performed retrospectively by independent researchers, who were unaware of the clinical and epidemiological characteristics of the patients included in the study.

### Statistical analysis

Continuous variables were described as mean ± standard deviation (m ± sd) or median and interquartile range, md (IQR), when the data did not show a normal distribution. The Kolmogorov-Smirnov test and analyses of the coefficients of kurtosis and asymmetry were performed to assess the normal distribution. The categorical variables were described as absolute and relative frequency distributions. For comparisons between the groups, independent Student’s t Test or Mann-Whitney non-parametric U-test was used for continuous variables and Pearson chi-square test (χ2) was used for categorical variables, with Yates correction. Kendall’s tau coefficient, a nonparametric hypothesis test, was used to verify the correlation between ST-Segment reduction and the angiographic patterns in dichotomous categorical variables. ROC (Receiver Operating Characteristic) curves were developed for the analysis of sensitivity/specificity of the ST-Segment regression based on the TIMI-flow and MBG scores. The positive likelihood ratio (LR) was calculated: LR (+): V = sensitivity / (1- specificity). Good diagnostic tests have a LR (+) >10 and its positive result has a significant contribution. The likelihood ratio for a negative LR (-) is a good indicator to rule out the diagnosis. Good diagnostic tests have LR (-) <0.1; calculated: LR (-) = (1- sensitivity) / specificity. The diagnostic odds ratio (DOR), is also a measure of accuracy, used to estimate the discrimination power and comparison of accuracy between the tests. The DOR was the ratio of the chance of positivity in the group with ST-Segment reduction >50% with the chances of positivity in the group without ST-segment reduction. A priori, a p-value <0.05 was considered statistically significant for all analyses, with bicaudal tests. The analyses were performed using the SPSS software, version-20 (IBM-SPSS Statistics, New York, USA)^®^

## Results

Patients of both genders, 70.2% of which were men were included; the median age was 58 years, IQR (50-66). The time from symptom onset to the primary care center was 220 minutes, IQR (140-345). The prevalence of risk factors and the patients’ characteristics in the basal period is reported in Table 1. Briefly, 60.7% were hypertensive; 29.8% had diabetes; 63.1% were smokers. In addition, 11% had a previous myocardial infarction and 4.3% had a previous stroke. The risk predictors used were recorded at the baseline period. Most patients were in low functional class by the Killip-Kimball classification:^8^**I** (73%), **II** (16.3%), **III** (2.2%), **IV** (8.6%); and had a low risk profile according to the prediction scores, TIMI-Risk:^9^ 3, IQR(2-5); GRACE^10^: 136, IQR (117-161), meaning a low risk profile.

**Table 1:** – Characteristics of the cohort studied in the basal period

Variables	All Patients (N = 2215)	Patients With ST-Reduction (n = 1511)	Patients Without ST-Reduction (n = 704)	P-value
Demographics				
Age, years; MD (IQR)	58 (50-66)	58 (50-66)	58 (49-66)	*0.62*
Male; (%)	70.2%	70.5%	69.6%	*0.41*
Body Mass Index, Kg/m^2^; MD (IQR)	26 (24-29)	26 (24-29)	26 (24-30)	*0.004*
**Hemodynamic Characteristics**				
Systolic Blood Pressure, mmHg; MD (IQR)	130 (115-150)	130 (116-150)	130 (110-150)	*0.098*
Diastolic Blood Pressure, mmHg; MD (IQR)	80 (70-93)	80 (70-94)	80 (70-93)	*0.23*
Heart rate, bpm; MD (IQR)	76 (66-90)	75 (66-88)	80 (68-95)	*<0.001*
Risk Factors				
Hypertension; (%)	60.7%	59.2%	64.5%	*0.01*
Diabetes mellitus*;* (%)	29.8%	28.1%	33.5%	*0.01*
Current smokers; (%)	63.1%	66.1%	57.5%	*<0.001*
Previous myocardial infarction; (%)	11%	9.9%	4.9%	*0.02*
Previous Stroke; (%)	4.3%	4.2%	4.1%	*0.98*
eGFR^**†**^, mL/min/1.73 m^2^; MD (IQR)	86 (67-108)	88 (70-110)	84 (63-106)	*0.015*
**Risk Scores**				
TIMI-Risk score; MD (IQR)	3 (2-5)	3 (2-5)	4 (2-6)	*<0.001*
GRACE-score; MD (IQR)	136 (117-161)	134 (118-157)	140 (115-171)	*0.004*
**Pivotal Times**				
Pain-Needle Time; MD (IQR) min.	220 (140-345)	220 (145-345)	220 (140-356)	*0.34*
Door-Needle Time; MD (IQR) min.	75 (45-135)	74 (45-130)	75 (44-150)	*0.057*
Time TNK^**‡**^-Cath-lab; MD (IQR) min.	740 (335-1380)	960 (405-1440)	410 (270-841)	<0.001

*Data on medical history, comorbidities and presentation time were largely derived from medical interviews. Demographic information and risk factors were reported by patients and trained staff reviewed the data during hospital admission. For comparisons between groups, the Mann-Whitney nonparametric test for continuous variables and χ2 test for discrete variables were used. The data are expressed as median (MD) and interquartile range (IQR), or number and percentage. eGFR^**†**^: glomerular filtration rate estimated by MDRD (Modification of Diet in Renal Disease); TNK^**‡**^: Tenecteplase; Cath-lab: catheterization laboratory.*

### Distribution of coronary flow according to TIMI / MBG

In the subjective evaluation of TIMI flow and MBG, the inter-observer agreement was 94%. The ARI distribution according to the TIMI-flow score (0-3) was: 21%; 3.6%; 14.4%; and 61% respectively. MBG grade was (0-3): 42%; 3.5%; 2.5%; and 52%, respectively, evaluated only in patients with TIMI-3 flow.

### Distribution of the infarct-related arteries

We observed the following distribution of the IRA: left anterior descending artery (40.3%); right coronary artery (35.3%); left circumflex coronary artery (6.8%); and branches of the main arteries (17.6%). The description of the IRA and the analysis of the regions involved in the ECG and their distribution, according to gender, can be seen in Table 2.

**Table 2:** – Description of the infarct-related artery and analysis of electrocardiographic regions with ST-segment elevation and its distribution according to gender

Infarct-related artery	All Patients N (%)	Men (%)	Women (%)
Anterior Descending Artery	893 (40.3)	41.9	36.4
Right Coronary Artery	782 (35.3)	33.5	39.6
Circumflex Coronary Artery	151 (6.8)	6.5	7.6
Left Coronary Artery (Trunk)	11 (0.5)	0.6	0.3
Posterior Descending Coronary Artery	8 (0.36)	0.3	0.5
Posterior Ventricular Coronary Artery	14 (0.63)	0.6	0.6
Left Marginal Coronary Artery	27 (1.21)	1.2	1.4
Diagonal Artery	15 (0.67)	0.6	0.9
Unidentified artery or other	335 (15.2)	15.4	13.1
**ST-Segment- elevation (ECG) **	**All Patients N (%)**	**Men (%)**	**Women (%)**
Anteroseptal wall	892 (40.3)	41.9	36.4
Anterior Wall	782 (35.3)	33.5	39.6
Extensive Anterior wall	151 (6.8)	6.5	7.6
Posterior wall	12 (0.5)	0.6	0.3
Inferolateral wall	49 (2.2)	2.1	2.4
Lateral wall	15 (0.7)	0.6	0.9
Unidentified or other	314 (14.2)	14.8	4.7

*The data are expressed as frequency and percentage (%). A team of independent experts reviewed the electrocardiographic findings. The agreement between observation pairs and the Kappa index was used as a measure of variability. The ECG reviewers were unaware of the patients’ baseline characteristics. ST-Segment elevation was measured 20 ms after point J. Infarction was considered as: anteroseptal (V1-V4); anterior: (V1-V6); extensive anterior: (V1-V6 + DI. aVL); inferior (DII, DIII, aVF); inferolateral: (DII, DIII, aVF + V5-V6); lateral (V5-V6). The infarct-related artery was identified according to the presence of thrombus, total occlusion or delayed anterograde flow.*

### Distribution of measurements by ECG criterion according to TIMI flow / MBG

The prediction of adequate coronary reperfusion using the ECG criterion (ST-Segment reduction >50%) showed a positive predictive value of 56% [95% CI (0.54-0.59)]; a negative predictive value of 66% [95% CI (0.62-0.70)]; sensitivity of 79% [95% CI (0.76-0.81)]; and specificity of 40% [95% CI (0.38-0.44)], a Positive likelihood ratio (LR +) of 1.32; and a negative likelihood ratio (LR -) of 0.52. The diagnostic odds ratio (DOR) was 2.55 (odds ratio of positivity in the group with ST-Segment reduction in relation to the chances of positivity in the group without ST-segment reduction). Thus, a weak positive correlation was observed between the ST-Segment reduction and the angiographic pattern of reperfusion, considering TIMI-3 and MBG-3, (r =0.21; *p<0.001*) as shown in Figure 2. We also highlight the behavior of the ECG criterion for arterial recanalization and its association with angiographic scores, stratified by gender, which were not significantly different (Table-3). Figure 3 shows the area under the curve (AUC) of 0.60 [95% CI (0.57-0.62)] in those with ST-Segment reduction and the angiographic pattern (TIMI-3 / MBG-3):[(**A**-overall; **B**-female; **D-**male)].

**Figure 2:** – Correlation between angiographic patterns (TIMI-3 and MBG-3) and the electrocardiographic criteria for reperfusion.

**Table 3:** – Correlation between ST-Segment reduction and TIMI-3 / Blush-3 scores, stratified by gender

Correlation Coefficient (Kendall index)					
Sex				ST Segment Reduction	TIMI-3 / Blush-3
Men	Kendall coefficient	ST-Reduction	Correlation Coefficient	1.0	0.203^*^
			N	1537	1506
		TIMI-3 / Blush-3	Correlation Coefficient	0.203^*^	1.0
			N	1506	1523
**Women**	Kendall coefficient	ST-Reduction	Correlation Coefficient	1.0	0.231^*^
			N	652	637
		TIMI-3 / Blush-3	Correlation Coefficient	0.231^*^	1.0
			N	637	643

*The correlation was significant at the level of 0.05 (bicaudal test).

**Figure 3:** – Area under the curve of patients with TIMI-flow 3 / Myocardial Blush Grade 3 and ST-Segment reduction for all patients and stratified by gender. A) Overall; B) Women; C) Rescue group; D) Men.

### Clinical characteristics of patients referred for rescue therapy

Patients were referred for rescue therapy after consensus of the local medical team, with a rate of 24.28%. The clinical-epidemiological characteristics between the rescue group and the elective angiography group is shown in Table 4. In the rescue group, the sensitivity and specificity was 59% and 53%, respectively, for the ST-segment reduction in the prediction of TIMI-3 / MBG-3. The AUC was 0.56 [95% CI (0.51-0.62)] as shown in Figure-3, (**C**-rescue group).

**Table 4:** – Clinical and epidemiological characteristics of patients in the elective angiography group and those referred for rescue angioplasty

Variables	Elective Angiography (n = 1677)	Rescue Angioplasty (n = 538)	P-value
**Demographic**			
Age, (years), m ± sd	58.26 ± 11.61	58.67 ± 11.53	0.47
Male, n (%)	1178 (71. 7%)	358 (66.5%)	0.03
**Risk Factors**			
Previous Myocardial Infarction, n (%)	218 (11.2%)	57 (10.6%)	0.75
Previous stroke, n (%)	108 (0.5%)	14 (2.6%)	0.05
Current smokers, n (%)	589 (35. 9%)	211 (39. 2%)	0.18
Hypertension, n (%)	678 (41. 3%)	186 (34. 7%)	0.01
Diabetes *mellitus, * n (%)	490 (27. 8%)	186 (34. 5%)	0.006
**Hemodynamic Variables**			
Systolic BP^**†**^, (mmHg), m ± sd	134.3 ± 27.56	130.91 ± 29.65	0.06
Diastolic BP^**†**^, (mmHg), m ± sd	82.78 ± 18.36	81.16 ± 19.50	0.11
Heart rate, (bpm). m ± sd	78 ± 17.55	81 ± 19.70	0.002

*Data are expressed as mean (m) ± standard deviation (sd) or percentage (%). Clinical demographic and risk factors were reviewed during hospital admission. BP^**†**^: Blood Pressure.*

## Discussion

A critical aspect of damage reduction associated with myocardial infarction is the guarantee of access to the emergency care, in which the identification of symptoms and the performance of the initial ECG are fundamental. With primary PCI unavailable in basic health units and in many hospitals, the drug-invasive strategy is recommended in STEMI.^2^The best method to obtain reperfusion has been widely debated, essentially skewed by the competitive perception regarding the possibilities of revascularization. It is well established that the best strategy is the one available within well-established deadlines, being indifferent within the first hours of the onset of ischemic symptoms. The trialist group demonstrated that in patients with pain within 6 hours, 30 deaths were prevented/1,000; between 7 and 12 hours, approximately 20 deaths/1,000 would be prevented.^11^Thus, the importance of the fast identification of ischemic symptoms and prompt care are highlighted. Consequently, even the primary PCI, as well as fibrinolysis, are well-established treatments and the benefits are maximized when treatment occurs as soon as possible.^12^A meta-analyses reported an increased risk of recent-onset heart failure, with a relative increase in the incidence per one hour-delay in reperfusion strategies.^13^

Our study was an attempt to provide a risk stratification through the interpretation of changes in bedside post-fibrinolysis ECG, determining which patients should be referred for urgent angiographic study and the ones that could be electively referred. In fact, the ECG plays a fundamental role in diagnostic accuracy and should be obtained as soon as possible. In the scenario of acute syndromes, reperfusion biomarkers, obtained at the bedside, are crucial for the stratification. Thus, the usefulness of the ST-Segment reduction criterion in predicting reperfusion has been used as a substitute outcome in some clinical trials. In a subgroup analysis from the DANAMI-2 cohort (DANish trial in Acute Myocardial Infarction-2), ST resolution after 4 hours was associated with higher reinfarction rates among patients receiving fibrinolytics, while it was not observed in patients receiving percutaneous treatment.^14^The time to obtain the ECG after fibrinolysis is variable. In the REACT study, an ST-reduction of 50% was defined at 90 minutes post-fibrinolysis, while the MERLIN study defined coronary reperfusion using the same criterion at 60 minutes. The mean time from symptom onset to PCI rescue was 414-minutes in the MERLIN study and 327 minutes in the REACT trial.^15,16^ Successful reperfusion was considered when the distal flow was restored to the infarct-related artery using the previously described TIMI-flow angiographic score.

In this context, the “open artery” hypothesis was postulated, indicating the prognostic impact if the artery was recanalized.^17^ Within this broad spectrum, Gibson et al.^18^introduced a refined method for the myocardial perfusion value: TIMI-flow and reperfusion therapy were considered angiographically successful when TIMI-3 was reached.^19,20^However, even when this score is reached, some patients have inadequate tissue perfusion and several mechanisms have been suggested, among others, distal embolization and the no-reflow phenomenon.^21-23^Subsequently, Hoffmann et al.^24^ disclosed the MBG concept with the angiographic index of the microvascular flow, since patients with TIMI-3 and normal MBG flow had lower mortality rates. The restoration of the coronary patency is not a guarantee of tissue perfusion; therefore, we understand that the presence of adequate MBG is a relevant prognostic characteristic and should be added to the TIMI- flow classification, commonly used to define successful angiographic reperfusion.^25-27^The abnormalities evaluated by MBG correlate with unfavorable ventricular remodeling, even after adjustment for the presence of TIMI-3 flow.^28-31^

Used as a reperfusion criterion, trials associated the persistence of ST-Segment elevation with the prognoses. In an interesting study, it was observed that in patients undergoing primary PCI, the ST-segment reduction did not predict long-term mortality.^32^Another study found no changes in the prognostic value of post-PCI ST-segment reduction in long-term follow-up for the main outcomes.^33^ In turn, the ST-Segment behavior within 60 minutes after successful primary PCI was also evaluated according to its association with lower short-and long-term mortality rates, with gradation of the ST-segment reduction (>70%; 30-70%; <30%). In this study, in the absence of ST-Segment reduction, patients with a lower chance of benefiting from early flow restoration of IRA were identified, probably due to microvascular damage and subsequent lower myocardial preservation.^34^ However, most of these studies enrolled patients submitted to primary PCI, not post-thrombolysis, and did not consider the MBG pattern as a defining variable of a better angiographic pattern of reperfusion. The presence of angiographically verified early resolution of the ST-Segment elevation post-primary PCI successfully identifies patients that are more likely to benefit from early restoration of flow in the IRA. It is also noteworthy that the TIMI-flow measures greatly overestimate the success in the primary PCI. In the post-fibrinolysis studies, the definition of angiographic success was characterized by the presence of TIMI 2 and 3. However, patients with TIMI-flow <3 show a worse clinical evolution, especially in the medium and long-term, when compared to the group of patients with TIMI-flow 3.

The accurate measurement of ST-Segment elevation is complex and could impair the simplicity of the model, especially in the emergency room. However, in most cases, accurate measurements are not necessary, as they can be easily obtained through the visual comparison of ECGs.^35^Several methods for evaluating ST-segment resolution are described, most of which include percentage of resolution; some use a single lead, while others measure the sum of deviations in multiple channels. In our notes, the detection of reperfusion using more rigorous criteria, such as complete resolution of the ST-Segment or reduction >70%, showed better specificity, but with low sensitivity and low predictive capacity. The prognostic accuracy of different methods in the evaluation of the ST-Segment after PCI was analyzed in the Controlled Abciximab and Device Investigation to Lower Late Angioplasty Complications (CADILLAC) study, demonstrating that the ST-segment analysis as absolute values in a single lead on the post-intervention ECG was at least equivalent, from the prognostic point of view, to more complex algorithms.^36^The evaluation of angiographic reperfusion as predicted by the ST-Segment changes was reported in our cohort. When we applied the correlation index between the TIMI-3 and MBG-3 criterion and the reperfusion, according to the ECG criterion, we obtained a value similar to that observed when only the angiographic classification of TIMI-3 flow was considered. Even after grouping and comparing patients with ischemic alterations in anterior wall vs. “non-anterior” infarction, the ST-Segment reduction >50% did not accurately predict the best angiographic patterns.

Even with effective therapies, there is still a lack of qualitative information available for stratification, specifically in the drug-invasive strategy model, where preliminary evaluation in centers and basic health units can be used to develop a better prognosis. The use of multivariate models as scores represents an interesting approach in prediction, superior to the obtained subjectively only by clinical impression. Tools that assist medical capacity by quickly and accurately assessing risk are therefore of great interest. Despite the existence of well-characterized predictors, risk estimation is challenging, due to complex profiles that require integration of variables and with calibration for local populations.^37^

In this cohort, we recorded 116 deaths (5.3%) and as expected, with a higher incidence in the rescue group (11.5% *vs*. 2.4%). Complications such as cardiogenic shock and severe electrical instabilities were the main causes of mortality, with patients with the highest lethality rates belonging to functional classification IV (Killip-Kimball). In the multivariate regression model, the Killip-Kimball class was the most powerful predictor, with an increasing risk of mortality with each degree of worsening of the category. Another important information to note is that the group that received earlier thrombolysis showed lower mortality rates, reinforcing the concept that early reperfusion attenuates the risk of complications, at least in the short term.

Ideally, a reperfusion indicator should be readily obtained and applicable mainly at the bedside. In our cohort, referral to the early angiographic study was possibly due to changes in the ST-Segment, because patients who did not have post-thrombolysis reduction were more rapidly referred to rescue therapy. In the group with ST-segment reduction >50%, the mean time between thrombolysis and the catheterization laboratory was 960 minutes *vs*. 410 minutes in the group without ST-segment reduction. However, this may have been a mistaken option, because 24.5% had TIMI-3 / MBG-3, characterizing a diagnosis of false-positive severity. In addition, 38.5% of the group with a reduction >50% showed an angiographic pattern of TIMI-flow <3, underestimating the real risk. In a recent study by our group, which assessed 104 patients, the analysis of the measurement of the QT interval and its dispersion in the 12 leads, and also only in the region with ST-segment elevation (named regional dispersion of the QT interval) pre and 60-minutes post-lysis, showed an increase in the regional dispersion of the QT interval (dQT-Reg) post-lysis in anterior wall infarctions in the cases with TIMI-3 / MBG-3 flow, with sensitivity and specificity of 93% and 71%, respectively. Although the study evaluated a small number of patients, these data suggest dQT-Reg as a promising instrument in the noninvasive identification of reperfusion.^38^

The ability to predict relief of ischemic symptoms during reperfusion is clinically recognized.^39^ However, this report is linked to idiosyncratic influences, as well as possible masking of symptoms by drugs (nitrates, analgesia, sedation). A serious problem identified in some centers is the inadvertent fibrinolysis by incorrect reading of ECGs, with reports that 5.7% to 11.0% of patients treated for STEMI did not have a myocardial infarction.^40^ In our cohort, we identified 7 cases that inadvertently received thrombolytics for the following causes: acute pericarditis, non-ischemic abnormalities of ventricular repolarization, and aortic dissection.

The implementation of early thrombolytic therapy remains a cornerstone to improve post-STEMI survival. However, as evidenced in our notes, the delay between symptom onset and admission to health units was prolonged, but there were no differences in the behavior of the ST-Segment after thrombolysis. In the multinomial regression, our data indicate the best angiographic results in patients referred early to hemodynamic study, regardless of the behavior of the ST-Segment. Therefore, we believe that the best post-thrombolysis strategy is the rapid referral to the angiography unit.^41^ Unfortunately the time available for these patients remains inadequate, possibly due to the serious logistical problems faced in large cities. For this reason, reducing these times remains a challenge in public health projects.^42^

### Limitations of the study

The limitations of the present study should be stated. This was an exploratory and single-center analysis. In addition, ECGs and angiographies were retrospectively analyzed. In clinical networks, risk stratification is only one of the parameters that determine the delay to the angiography. In the STREAM study,^3^ the mean time until the angiography was 2.2 hours for patients who required urgent intervention and 17 hours for the remaining 64%. However, our findings indicate higher times, especially for the rescue group, maybe due to difficulties in transporting patients to the hemodynamic center (ambulance not immediately available). However, this is a common situation of public services in a large city. As a last point to be mentioned, our study did not specifically test ECG performance for reperfusion but analyzed the recommended ECG guidelines criteria.

## Conclusion

Our results suggest that the ST-Segment reduction, analyzed as recommended, could not accurately identify patients with adequate angiographic reperfusion, based on TIMI-flow and MBG scores. We believe that an early angiographic study offers the opportunity to identify fibrinolysis failures. Thus, in the absence of better biomarkers, even patients with apparently successful reperfusions should be referred to the hemodynamics laboratory as soon as possible, aiming to ensure adequate coronary flow, considering the macro and microvascular aspects.
